# Retrospective study on the incidence of envenomation and accessibility to antivenom in Burkina Faso

**DOI:** 10.1186/s40409-016-0066-7

**Published:** 2016-03-16

**Authors:** Sandrine Gampini, Sonia Nassouri, Jean-Philippe Chippaux, Rasmané Semde

**Affiliations:** Ministère de la Santé, Direction générale de la pharmacie du médicament et des laboratoires, 03 BP 7009 Ouagadougou 03, Kadiogo Burkina Faso; UMR 216, Mère et enfant face aux infections tropicales, Institut de Recherche pour le Développement, Cotonou, Bénin; Université Paris Descartes, Sorbonne Paris Cité, Faculté de Pharmacie, Paris, France; Département des Sciences Pharmaceutiques, Université de Ouagadougou, Ouagadougou, Burkina Faso

**Keywords:** Envenomation, Snakebite, Antivenom, Burkina Faso

## Abstract

**Background:**

Snakebite is a common neglected public health issue, especially in poor rural areas of sub-Saharan Africa, Asia and Latin America. Passive immunotherapy with safe and effective antivenom is the only approved treatment for it. This study aimed to determine the incidence of snakebites, and to assess the availability and accessibility of antivenoms, from 2010 to 2014, in Burkina Faso.

**Methods:**

The assessment of snakebite cases managed in all health facilities from 2010 to 2014 was performed from the Statistical Yearbook of the Ministry of Health. Antivenom consumption data were collected from the drug wholesalers established in Burkina Faso.

**Results:**

Snakebites are among the five leading causes of consultations in health districts. From 2010 to 2014, 114,126 envenomation cases occurred in Burkina Faso, out of which 62,293 (54.6 %) victims have been hospitalized resulting in 1,362 (2 %) deaths. The annual incidence and mortality were respectively 130 bites and 1.75 deaths per 100,000 inhabitants. The amount of antivenom sold by wholesalers were 5,738 vials with a total cost of US$ 539,055 (annual average = US$ 107,811). The high cost of these antivenoms (between US$ 42 and 170 per dose according to brand) limited their use by rural people, the main victims of snakebites, whose income is insufficient. Thus, only 4 % of patients received antivenom treatment over the past five years. The price of antivenom was reduced in 2015 to US$ 3.4 by a public drug wholesaler.

**Conclusion:**

The study confirmed the high burden of snakebites in Burkina Faso. To better manage envenomation, Burkina Faso implemented a strategy consisting in seeking affordable sources of antivenom supply of good quality and innovative mechanisms of subsidy.

## Background

Although snakebite envenomation is considered a particularly important and frequent public health hazard in several countries, it remains largely neglected. It affects mainly rural areas of tropical and subtropical countries, particularly sub-Saharan Africa where the management is inefficient. Besides the cost of antivenom, sometimes equivalent to several months of a family income, the lack of standardized treatment protocol due to insufficient training of health personnel as well as underestimation of the incidence and severity of snakebites make difficult the implementation of a management strategy both for prevention and treatment [1].

In Burkina Faso, the different snake species belong to six families, among which Elapidae and Viperidae are the most venomous [2]. Passive immunotherapy with safe and effective animal-derived antivenom is the only approved treatment. However, its availability is questioned because of its high cost and poor demand from victims and health professionals who do not use it [3].

A recent meta-analysis of the literature estimated the average annual snakebite incidence and mortality in Burkina Faso, respectively, at 7,341 ± 1,260 envenomations and 343 ± 73 deaths [4]. Those figures, useful for a basic approach to the implementation of snakebite management in health services, are imprecise and subject to biases according to the representativeness of the studies used for the assessment.

Burkina Faso has a pyramidal health system in three levels with a national health statistic service that provides centralized epidemiological data. The notification of snakebites at different levels of the health pyramid is operational since 2010. We collected data provided by the Ministry of Health on all patients treated in health facilities in the country aiming to evaluate the annual incidence and mortality from snakebites in Burkina between 2010 and 2014. The present findings may serve as an indicator to define an appropriate management strategy. We also evaluated the availability of antivenom during this period in order to propose some improvements in its accessibility.

## Methods

Burkina Faso is divided into 13 health regions. General population data were provided by the National Institute of Statistics and Demography (INSD), the central organ for official statistics production in Burkina Faso (http://www.insd.bf/n/). Methods and results of the last census in 2006 are accessible at http://www.insd.bf/n/nada/index.php/catalog/23 (in French).

Snakebites treated in health facilities of the country from 2010 to 2014 have been notified according to levels of the health pyramid by the Ministry of Health in the sanitary statistical yearbooks. Some of these documents are online (http://www.cns.bf/IMG/pdf/annuaire_ms_2012.pdf; http://www.cns.bf/IMG/pdf/annuaire_sante_2013.pdf), while others are available in the archives of the Ministry of Health in Ouagadougou, Burkina Faso.

Each annual report specified the incidence and mortality of major diseases diagnosed in health facilities of the country, including snakebites, whose declaration is mandatory since 2010. The data were presented by gender, age group and geographic regions. Moreover, outpatients (not hospitalized) and patients undergoing a hospital stay of at least 24 h were presented separately.

The antivenom consumption data were collected from the main drug wholesalers in the country: Ubipharm, CAMEG, Laborex, DPD and Tedis Pharma. Each wholesaler was asked about orders and sales of antivenom between 2010 and 2014 and provided the corresponding data.

Statistical tests used were Pearson’s *χ*^2^ test with Yates’ continuity correction and the Pearson correlation test. Excel® statistical tools were used to perform the analysis. The statistical significance and confidence intervals were *p* = 0.05.

## Results

### Snakebite incidence and mortality

The total number of snakebite cases recorded from 2010 to 2014 was 114,126 with an average annual incidence of 136 ± 9 bites per 100,000 people (Table 1).

**Table 1:** Annual incidence of snakebites in Burkina Faso, 2010–2014

Years	Population	Number of snakebites	Incidence per 100,000 population
2010	15,730,977	22,662	144
2011	16,248,558	22,706	140
2012	16,779,207	23,343	139
2013	17,322,796	20,636	119
2014	17,880,386	24,779^a^	143^a^
Mean	16,792,385	22,337	136

^a^Unconsolidated data, not included in the calculation of the mean

Table 2 specifies the number of snakebites treated in the various health facilities. Outdoor patients corresponded to snakebite victims treated as outpatients and whose health did not require hospitalization. Hospitalized patients were envenomated people that required more than 24 h of hospitalization and showed quite severe symptoms (not detailed in the reports). According to the Ministry of Health, snakebites, nationally and regardless of severity, appeared as one of the top five causes of consultations in health facilities of first level, with an annual average of 13,117 cases, after malaria (over 316,000 cases a year), acute respiratory infections (nearly 55,000 annual cases) and anemia (14,000 per year), but before diarrhea (10,300 cases annually) coming in fifth.

**Table 2:** Snakebite distribution according to the severity, 2010–2014

Years	Outdoor patient	Hospitalized patients	Total snakebites	Total deaths
2010	7,942	14,720	22,662	346
2011	7,235	15,471	22,706	307
2012	7,883	15,460	23,343	237
2013	7,543	13,093	20,636	283
2014	21,230^a^	3,549^a^	24,779^a^	189^a^
Mean	7,651	14,686	22,337	293

^a^Unconsolidated data, not included in the calculation of the mean

**Fig. 1:**
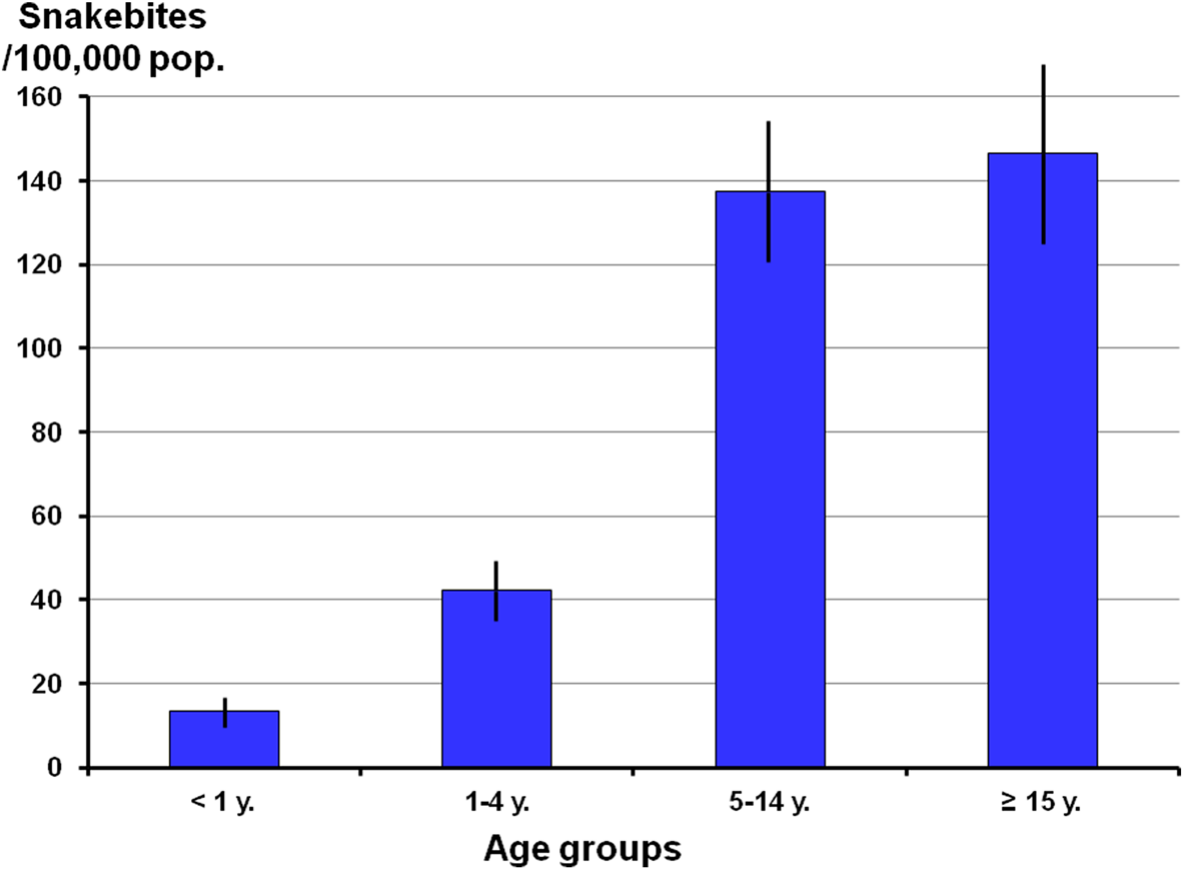
Annual specific incidence of snakebites per 100,000 people according to the age group

**Fig. 2:**
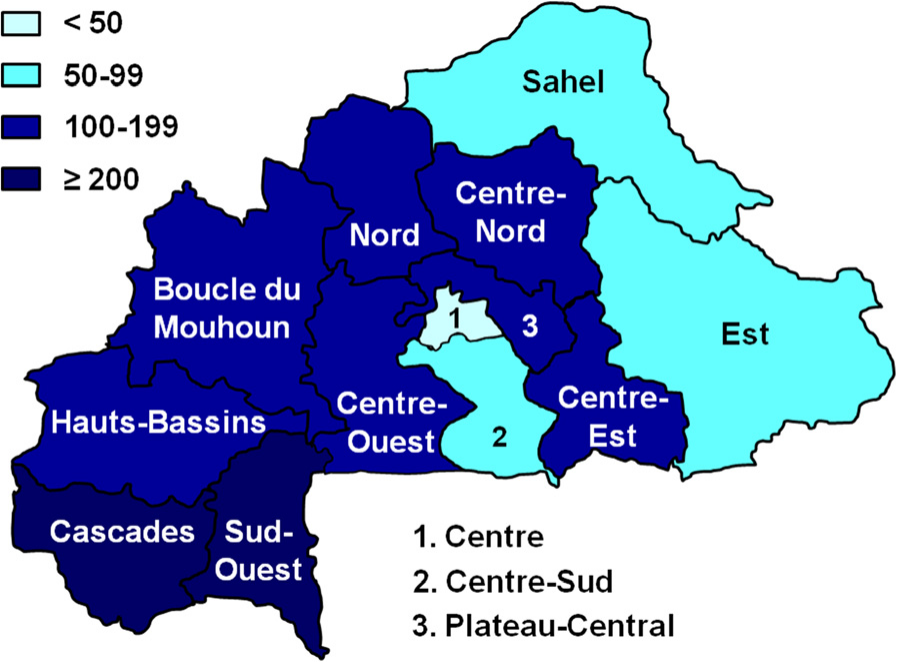
Geographic distribution of snakebites in Burkina Faso (annual mean incidence per 100,000 population)

The sex ratio was 1.1 (males/females) for patients aged 15 and older. The age distribution (Fig. 1) showed a significantly higher incidence in patients older than five years (*χ*^2^ = 1942; *p* < 10^−5^). The geographical distribution is shown in Table 3 and Fig. 2. There was no significant correlation between the incidence and population density (*r*^2^ = 0.48; *p* > 0.065). Between 2010 and 2014, health services reported 1,362 (1.2 %) deaths, 293 on average each year. Annual mortality was between 1.13 and 2.06 deaths per 100,000 inhabitants (mean = 1.75). The average hospitalization rate was 66 %, not including 2014 for which data were not yet consolidated.

**Table 3:** Geographical annual incidence of snakebites (per 100,000 population)

Regions	Population	Density	Incidence
Boucle du Mouhoun	1,700,424	50	184 ± 29
Cascades	674,553	36	205 ± 10
Centre	2,280,653	795	24 ± 2
Centre-Est	1,363,871	93	145 ± 82
Centre-Nord	1,438,149	74	114 ± 6
Centre-Ouest	1,407,877	65	171 ± 28
Centre-Sud	752,505	66	72 ± 9
Est	1,489,004	32	99 ± 1
Hauts-Bassins	1,806,821	71	164 ± 12
Nord	1,401,682	78	131 ± 91
Plateau-Central	818,568	95	148 ± 18
Sahel	1,176,768	33	81 ± 8
Sud-Ouest	740,128	45	295 ± 19
Total – Burkina Faso	17,051,002	62	130 ± 10

### Antivenom availability

In Burkina Faso, as occurs with other drugs and in accordance with regulations, antivenom is sold by licensed wholesalers. The number of vials sold between 2010 and 2014 was 5,738 with a total cost of US $ 539,055 (Table 4).

**Table 4:** Evolution of the number of doses and cost of antivenom, 2010–2014

Years	Number of doses	Overall cost (US$)	Mean of dose cost
2010	598	74,827	125
2011	846	105,859	125
2012	685	79,770	116
2013	1,100	82,896	75
2014	2,509	195,702	78
Mean of annual cost	1,148	107,811	104

Two types of antivenom were available. The FAV-Africa (Sanofi-Pasteur, France) and the polyvalent serum West Africa EchiTAb-Plus-ICP (Instituto Clodomiro Picado, Costa Rica). They respectively accounted for 63.9 % and 36.1 % of sales and price transfer to public and private structures were US$ 170 and 42, i.e., about US$ 200 and 50 to the patients.

Less than 4 % of patients received antivenin treatment over the past five years.

## Discussion

The main objective of this study was to better estimate the quantitative and qualitative demand for antivenom in Burkina Faso [5]. Firstly, we wanted compare crude data obtained from the epidemiological reporting system of the Ministry of Health and the assessment based on the meta-analysis from medical and scientific publications [4]. Secondly, our goal was to provide updated and accurate data in order to define the necessary amount of antivenom and the most relevant geographic location to offer it.

Like any retrospective study, our study presented some limitations [6]. Reported data did not always contain relevant and useful aspects for the implementation of strategies for the prevention or management of snakebites. Moreover, although the collection tools significantly improved between 2010 and 2014, health statistics did not provide useful information to health authorities regarding, for example, seasonality of snakebites or treatment seeking behavior of snakebite victims [1].

Another limitation was the lack of data from private health centers. Most religious hospitals contributed in the epidemiological reporting system and purchased antivenom from the wholesalers interviewed. In contrast, small private clinics did not fit that framework. However, their medical coverage was poor and they rarely treated snakebites, preferring to refer them to the nearest hospital.

Most epidemiological data confirmed the results obtained elsewhere [4]. Snakebites occurred in active rural populations, mainly men, although in Burkina Faso, it seems that the number of women victims of snakebites (47 % of adults bitten by a snake) was higher than expected, as already stated by Somé et al. [7]. Indeed, generally in Africa, the proportion of women bitten by a snake is low [4]. The reasons are not known but one can make the assumption that women are less often present in areas at risk of bites. Indeed, snake populations vary according to the biotopes and particularly type of plantations, which may explain the difference, since men and women are not working in the same field or at the same hours [2, 7]. Another hypothesis is that women consulted less frequently health centers than men in our study.

Although there is a higher incidence in rural areas, particularly in the south and west of the country, there was no significant correlation between the incidence of bites and population density. Nevertheless, the incidence of snakebites was significantly lower in highly urbanized areas like the Center, where the capital Ouagadougou is located, with an average annual incidence of snakebites of 23 per 100,000 population (Table 3; Fig. 2) compared to rural districts of South-West Burkina Faso where the incidence can reach 300 per 100.000 population. This suggests that the distribution of venomous snakes involves populated areas including towns [8].

Although the incidence of snakebites was more than the double of previous estimates, it is likely to remain underrated [4]. For example, many patients preferred to consult a traditional healer as indicated by a previous study in the southwest of Burkina Faso in 2002 [7]. In addition, several patients bitten by Elapidae snakes die before reaching health centers due to a long delay between the bite and admission to hospitals [9].

The available data did not allow the assessment of envenomation severity, except when case fatality rate reached 1.2 % of the bites (2.2 % of hospitalized patients). Outpatients (51,833 patients between 2010 and 2014) were supposed to have either asymptomatic bites or mild envenomation. In 2014, the number of snakebites increased considerably (over 2,500 cases above the average of other years), but also showed an inverted distribution between outdoor patients and hospitalized patients. The 2014 data have not yet been consolidated and we could not find any explanation for the difference compared to other years. It may result in a better management of snakebites in the peripheral health facilities, perhaps through better distribution of antivenom, which resulted in avoiding referral to the hospital. Note that the number of antivenom doses used in 2014 was also higher than in other years. Unfortunately, we do not know at what level of the health pyramid these doses were administered. Moreover, even if their number was higher, it did not reach the theoretical number of doses corresponding to the number of registered cases.

Indeed, conditions, constraints and cost of hospitalization were strongly dissuasive for patients with no or mild envenomation or for those whose symptoms did not force them to resort to hospitalization. Consequently, it is possible to deduce from the number of hospitalized patients the minimal number of envenomed patients – on average 14,700 per year – who required specific medical care, including antivenom. This could be compared to the estimated annual incidence of envenomation by Chippaux [4], which was half of the present estimation (7,341 ± 1,260). In contrast, the estimated number of deaths by Chippaux [4] (343 ± 73) was more close to our results, perhaps because the reporting of deaths is more accurate than estimates of snakebites.

The total number of sold antivenoms was very low considering the incidence. Applying the treatment protocol recommended by the African Society of Venimology would require more than 25,000 doses each year in order to reduce 90 % of mortality and prevent many disabilities by limb amputation following necrosis caused by Viper bites [10]. However, very few patients (3.9 %) received antivenom treatment. Yet it was not clear how many doses each patient received and it is possible that some of them suffered complications (death, amputation, permanent disability) as the result of uncontrolled envenomation.

The reasons for the poor adherence to antivenom treatment could be explained by their limited accessibility because of numerous factors already described, namely: high cost, lack of training of medical personnel, inaccurate prediction of storage and distribution due to the absence of relevant epidemiological data etc. [1, 3, 10]. The price of antivenom was the same, regardless of the health institution that acquired it, resulting in a similar cost to all health centers. However, as in most sub-Saharan countries, the cost of care is – so far – fully supported by the patient and his family.

The high cost of antivenoms considerably limited their access because the low income of the rural population – the main victims of snakebites – prevented them to adhere to an unaffordable treatment.

In the face of this situation, when negotiating the prices of essential medicines with the Ministry of Health and civil society, the public wholesaler (Centrale d’Achat des Médicaments Essentiels Génériques et des Consommables Médicaux du Burkina Faso, CAMEG) agreed in 2015 to cut the price of antivenom to US$ 3.4, since a recent decision on health policy engages the government to support the price difference through a subsidy. This modification in the health policy should not change the price of care between public and private health facilities because all health facilities can provide antivenom purchased from CAMEG. Now, patients buy the antivenom at 2,000 CFAF (about US $ 3), which represents less than 5 % of its actual price, the rest is paid by the government. This new policy should greatly facilitate access to good quality care.

## Conclusion

The notification of snakebites is operational in Burkina Faso since 2010, which helped in clarifying and mapping their incidence and mortality. With over 20,000 snakebites, of which nearly 15,000 envenomations, treated in health facilities, and about 300 deaths reported every year, this public health issue remains a concern. Rural populations, particularly young active adults, are much more exposed than urban people.

The underutilization of antivenom by health centers is obvious but can be reverted. Knowing better the actual demand and provided that health staff is appropriately trained, it will be possible to switch the vicious cycle of inaccessibility of antivenoms [1]. The government subsidy will offer a realistic and probably effective aid in this matter.

### Ethics approval

This study was based on mandatory notifications made by the health centers to the Ministry of Health and did not require ethical clearance.
